# Does Whole-Body Vibration Treatment Make Children’s Bones Stronger?

**DOI:** 10.1007/s11914-020-00608-0

**Published:** 2020-07-21

**Authors:** Diana Swolin-Eide, Per Magnusson

**Affiliations:** 1 Department of Pediatrics, Institute of Clinical Sciences, Sahlgrenska Academy, University of Gothenburg, Gothenburg, Sweden; 2 Region Västra Götaland, Sahlgrenska University Hospital, Department of Pediatrics, The Queen Silvia Children’s Hospital, Gothenburg, Sweden; 3 Department of Clinical Chemistry, and Department of Biomedical and Clinical Sciences, Linköping University, Linköping, Sweden

**Keywords:** Bone mineral density, Pediatric, Mechanical oscillation, Rehabilitation, Physical activity, Skeleton

## Abstract

**Purpose of Review:**

To summarize the last 10 years of literature regarding the effects of whole-body vibration (WBV) on bone in children, and if WBV results in increased bone acquisition.

**Recent Findings:**

WBV intervention appears to be a safe intervention with beneficial effects on bone mass in some diseases and syndromes, but there is still low evidence for WBV in clinical practice. The positive effects on muscle strength, balance, and walking speed are more conclusive. One of the takeaways of this review is that well-trained individuals may not further improve bone mass with WBV; thus, interventions are more beneficial in pediatric individuals with Down syndrome or severe motor disabilities with low bone mass and reduced activity levels.

**Summary:**

WBV appears to be a safe non-pharmacological anabolic approach to increase bone mass in some pediatric populations; however, longer (> 6 months) and larger prospective studies are needed to elucidate the efficacy of WBV on bone health in young individuals.

## Introduction

Bone mass increases gradually under healthy conditions during childhood and reaches a plateau (a.k.a. peak bone mass) in early adulthood, which serves as a “bone bank” for the remainder of life. Longitudinal growth and bone modeling during childhood is a complex process of both resorption and formation that is necessary for skeletal growth and it has been shown by a number of studies that physical activity increases bone formation and bone acquisition [1, 2].

Whole-body vibration (WBV) was initially developed in the 1970s to prevent loss of muscle and bone mass in cosmonauts during prolonged spaceflights [3]. The underlying mechanism of concept that WBV could increase bone mass relates to the mechanostat theory; that is, bone adapts its strength to mechanical forces that are mostly imposed by muscle [4••, 5]. An early study in sheep by Rubin et al. [6] showed that low-level mechanical stimulation resulted in a strong anabolic response through increased bone formation in trabecular bone after 1 year. These results were further strengthened by experimental studies in rats where the anabolic activity on bone, suppressed by disuse, was normalized by mechanical stimulation [7]. As a development of these positive results in animal models with anabolic effects on bone, WBV has been developed for humans as an anabolic option to improve bone mass. WBV could be an alternative to replace and/or complement regular physical activity. Intervention including WBV has also shown a number of metabolic effects [8, 9] and, in addition, WBV increases muscle power and muscle strength [10]. The mechanical stimulation from WBV affects bone cells, such as osteocytes, which results in altered expression of Wnt-signaling proteins, e.g., sclerostin, resulting in increased bone mass [11••, 12••].

**Fig. 1:**
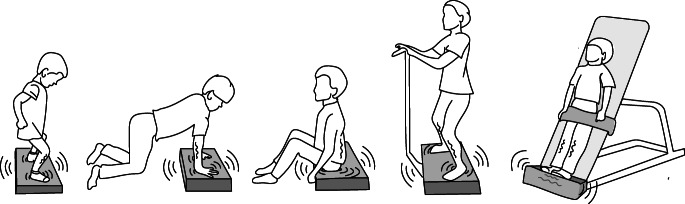
Schematic examples of different uses of WBV platforms

WBV has, therefore, received increasing attention as a treatment option including pediatric patient populations with individuals in the phase of bone acquisition. Young individuals with a broad variety of diseases which leads to poor bone health could be considered for WBV, hopefully without side effects, as a non-pharmacological anabolic approach to increase balance, neuromuscular function, and bone mass [13••]. The literature presents various WBV platforms with vibration strategies and as scientists should be able to reproduce the study and data, there was an early need for an international consensus on how to report data and how to describe the vibration intervention by the International Society of Musculoskeletal and Neuronal Interactions [14••]. Vibration platforms do not only differ with respect to vibration parameters such as frequency, amplitude, and acceleration, but also in the type of mode they vibrate, that is, side-alternating by oscillation around a horizontal anteroposterior central axis, or synchronous vibration with uniform acceleration and peak-to-peak displacement for the entire surface. Figure 1 demonstrates different uses and positions of WBV platforms. For the included studies in this review, the different settings of vibration parameters and intervention details are presented in Table 1.

**Table 1:** Summary information of pediatric WBV studies (during the last 10 years) with major bone outcomes

Reference	Study design	N	Age (years) Gender	Type of WBV	Intervention details	Major bone outcomes
Healthy children and adolescents						
Binkley et al. [15] (2014)	Randomized controlled trial	20	6–10 M + F	WBV on upper limbs HMMS 14 Hz, 0.75 g; LMMS 29 Hz, 0.30 g	HMMS 2 min and LMMS 5 min 3 days/week for 12 weeks	Increased trabecular BMD in the radius
Harrison et al. [16] (2015)	Randomized controlled trial	36	9–12 M	Two WBV standing platforms, high or low magnitude, high > 2 g or low < 1 g	On either 1, 3 or 5 successive days	Bone turnover markers PINP and CTX increased after 8 days
Gomez-Bruton et al. [17] (2018)	Randomized controlled trial	51	14 ± 2 M + F	WBV standing platform 38 Hz, 4 mm, 12 g	15 min, 3 days/week for 6 months	No effect on bone strength or bone structure
Muscle degenerative disorders						
Bianchi et al. [18] (2013)	Randomized controlled trial	21	9 ± 4 M	Low-magnitude high-frequency	10 min/day for 1 year	Increased BMD
Söderpalm et al. [19] (2013)	Observational prospective trial	6	6–12 M	WBV standing platform 16–24 Hz, 4 mm, 2.1–4.6 g	2–3 times/week for 3 months	No effect on bone mass
Petryk et al. [20] (2017)	Observational prospective trial	5	6–22 M	WBV standing platform Low-magnitude high-frequency, 30–90 Hz	10 min/day for 6 months	Uncertain effects on cortical and trabecular parameters
Severe motor disabilities						
Ruck et al. [21] (2010)	Randomized controlled trial	20	6–12 M + F	WBV standing platform 12–18 Hz, 4 mm, 2.6 g	9 min/school day for 6 months	No effect on bone mass
Afzal et al. [22] (2014)	Crossover pilot study	14	3–20 M + F	WBV standing platform Low-magnitude, 30 Hz, 0.3 g	20 min/day, 5 days/week for 12 months	Positive effects on bone mass, especially in spine BMD *Z*-score
Gusso et al. [23] (2016)	Clinical trial without control group	40	11–21 M + F	WBV standing platform 12–20 Hz	9 min, 4 days/week for 20 weeks	Positive effects on bone mass (total body, lumbar spine and lower limbs)
Kilebrant et al. [24] (2015)	Observational, prospective	19	5–16 M + F	WBV standing platform 40–42 Hz, 0.2 mm	5–15 min twice/week for 6 months	Positive effects on bone mass for total body BMD
Reyes et al. [25] (2011)	Randomized controlled trial	65	6–9 M + F	WBV device on elbows and knees Low-magnitude high-frequency, 60–90 Hz, 0.1 mm, 0.3 g	5 min/day, 7 days/week for 6 months	Positive effects on regional bone mass, ultradistal radius
Stark et al. [26] (2010)	Retrospective, home-based WBV and other training	78	Mean 10 M + F	WBV standing platform 5–25 Hz, 0–3.9 mm	6-month home-based training	Positive effects on bone mass
Wren et al. [27] (2010)	Randomized controlled trial	31	6–12 M + F	WBV standing platform 30 Hz, 0.3 g	10 min/day for 6 months	Positive effects on bone mass, especially in cortical bone
Osteogenesis imperfecta						
Hoyer-Kuhn et al. [28] (2014)	Retrospective, home-based WBV, and other training	53	2–25 M + F	WBV standing platform 15–20 Hz, 0–7.8 mm, 3.5–6.3 g	Twice daily (3 × 3 min) for 6 months	Positive effects on bone mass. Total body without head BMD increased.
Högler et al. [29] (2017)	Randomized controlled trial	24	5–16 M + F	WBV standing platform 20–25 Hz, 2–6 mm	Twice daily (3 × 3 min) for 5 months	No effect on bone mass
Other groups						
*Down syndrome* Matute-Llorente et al. [30] (2016)	Randomized controlled trial	25	12–18 M + F	WBV standing platform 25–30 Hz, 2 mm, 2.5–3.6 g	3 times per week with 10 repetitions of 10–20 s, for 20 weeks	Positive effects on bone mass
*Obesity* Tubic et al. [12••] (2019)	Randomized controlled trial	30	7–17 M + F	WBV standing platform 16–24 Hz, 4 mm, 2.1–4.6 g	7–10 min, 3 days/week for 3 months	No effect on bone mass, but effects for serum sclerostin
*Overweight* Erceg et al. [31] (2015)	Randomized controlled trial	20	8–10 M	WBV standing platform 30–40 Hz, low-high, 1.9–6.2 g	3 times per week with 10 repetitions of 30–60 s, for 10 weeks	No effect on bone mass
*Anorexia nervosa* DiVasta et al. [32] (2017)	Randomized double-blind trial	41	13–21 F	WBV standing platform LMMS 32–37 Hz, 0.3 g	10 min/day during hospitalization of 5 days	Prevents a decline in bone turnover during bed rest
*Idiopathic scoliosis* Lam et al. [33] (2013)	Randomized controlled trial	149	15–25 F	WBV standing platform LMMS 32–37 Hz, 0.3 g	20 min/day, 5 days/week for 12 months	Positive effects on bone mass
*Thalassemia* Fung et al. [34] (2012)	Longitudinal crossover pilot trial	18	10–18 M + F	WBV standing platform LMMS 30 Hz, 0.3 g	20 min/day for 6 months	Positive effects on bone mass
*Hemophilia* El-Shamy [35] (2017)	Randomized controlled trial	30	9–13 M	WBV standing platform 30–40 Hz, 2–4 mm	15 min/day, 3 days/week for 12 weeks	Positive effects on bone mass
*Thermally injured* Edionwe et al. [36] (2016)	Randomized controlled trial	19	11–13 M + F	WBV standing platform 30–40 Hz, 2–4 mm	12–15 min/day, 5 days/week for 6 weeks	Stabilizing the decline in bone mass
*Crohn’s disease* Leonard et al. [37] (2016)	Randomized controlled trial	138	8–21 M + F	WBV standing platform LMMS 30 Hz, 0.3 g	10 min/day for 12 months	Increased vertebral trabecular BMD, but inconsistent effects on axial and appendicular trabecular volumetric BMD

*F*, female; *HMMS*, high-magnitude mechanical stimulation; *LMMS*, low-magnitude mechanical stimulation; *M*, male

Some studies have been reported about the effects of WBV on bone mass in children and adolescents; however, the potential effects and protocols with optimal vibration parameters are still uncertain. This review aimed to assess the literature during the last 10 years regarding the effects of vibration treatment on bone in pediatric populations. Research publications were identified by searching PubMed with the applied search string (filter 10 years): vibration AND (bone OR skeleton OR BMD OR osteoporosis) AND (children OR adolescents OR pediatric) AND human, until February 2020 without language restrictions. A total of 156 publications were found with this search strategy. Table 1 summarizes the selected original articles during the last 10 years regarding the effects of WBV intervention protocols in pediatric populations.

## Safety of WBV

Most studies covered by this review did not report serious adverse events of WBV, which is in conjunction with other reviews on this topic [38, 39, 40••, 41••]. Söderpalm et al. [19] studied WBV exercise (2 to 3 times a week, 3 months) in patients with Duchenne muscular dystrophy (DMD). The circulating levels of creatine kinase did not change over the study period, thus indicating that WBV exercise, at this magnitude, was well tolerated and did not induce further skeletal muscle damage. No serious adverse events were reported in the meta-analysis by Saquetto et al. [40••] comprising 176 patients with cerebral palsy (CP) from 6 studies, and WBV was considered well tolerated in these cohorts although that potential long-term risks require more research. In a study with adult women, lower leg itching and erythema were reported [42]. Another study in children with CP reported that 80% of the participants experienced redness of the feet after the first treatment session [21]. As reported by the review by Bell et al. [41••], many studies do not provide information on adverse events; however, we would like to highlight the importance of reporting adverse events and all negative side effects in future clinical WBV studies, since this has to be taken into account in future clinical practice guidelines.

## Effect of WBV on Healthy Children and Adolescents

There are only few studies concerning WBV intervention and the effect on bone mass in healthy young individuals. A randomized controlled trial in healthy pre-pubertal children with high and low mechanical stimulation vibration for 12 weeks increased trabecular bone mineral density (BMD) in the forearm [15]. Rapid effects of WBV on bone remodeling have been studied in healthy pre-pubertal boys by using biomarkers of bone turnover. After 5 consecutive days of WBV training (applying two platforms with high and low-magnitude vibration), it was demonstrated that the bone formation marker PINP (i.e., type I procollagen intact amino-terminal propeptide) increased by 25% and the bone resorption marker CTX (i.e., carboxy-terminal cross-linking telopeptide of type I collagen) by 10%; however, no effect was found for serum osteocalcin, osteoprotegerin, or sclerostin [16]. The authors suggested that irrespectively of the magnitude of vibration, the healthy growing bone tissue does have the capacity to respond quickly to WBV training. The review by Marin-Puyalto et al. [43••] concluded that interventions with WBV appears to be more effective in increasing bone mass in young individuals with compromised bone mass in comparison with postmenopausal women. No effect was found on bone strength or structure in a study with healthy adolescent swimmers who performed swimming training and WBV intervention three times a week during a 6-month study period. These authors suggested that WBV intervention was not intense enough to achieve positive effects on skeletal strength [17].

## Muscle Degenerative Disorders

Both Duchenne and Becker muscular dystrophies are X-linked progressive neuromuscular disorders caused by loss-of-function mutations in the gene *DMD* coding for the protein dystrophin. Affected patients with DMD have their first signs of muscle weakness during childhood. Becker muscular dystrophy is usually milder and more varied. Poor bone health is common in patients with DMD, and long-term corticosteroid treatment further increases the risk for osteoporosis and fragility fractures [41••, 44].

Bianchi et al. [18] showed in a small pilot study that BMD increased in spine, total body, and femoral neck in patients with DMD, which is in contrast to another small study in which no effects were found on bone mass, muscle strength, or biomarkers of bone turnover [19]. In another small study, Petryk et al. [20] observed uncertain effects on cortical and trabecular parameters.

Most WBV studies in patients with DMD are small observational investigations, which makes it difficult to draw any significant conclusions regarding the efficacy of WBV in patients with muscle degenerative disorders. However, WBV interventions appear to be well tolerated in patients with muscular dystrophies; hence, larger controlled trials are needed to establish potential benefits of WBV before any clinical implications can be made.

## Severe Motor Disabilities

Fragility fractures, as a consequence of reduced BMD, are common complications in children with severe motor disabilities such as CP and Rett syndrome [45]. The prevalence rate for fragility fractures is nearly 20% in non-ambulatory children and young adults with CP [46]. There is, therefore, an increasing interest in WBV as a non-pharmacological anabolic approach in children with severe motor disabilities to increase neuromuscular function, balance, and bone mass. For this review, we found 8 intervention studies reported in PubMed (during the last 10 years) about WBV therapy in children with severe motor disabilities.

In a study with 16 patients with CP, aged 9 years, spasticity was reduced and ambulatory function improved after 8 weeks of WBV intervention; however, bone parameters were not investigated [47]. A randomized controlled pilot study with WBV treatment in 20 children with CP detected improved mobility function but did not detect any positive effect on bone tissue after 6 months of treatment [21]. However, positive effects on cortical and trabecular bone have been demonstrated in a number of studies on patients with CP and Rett syndrome [22–27]. Saquetto et al. [40••] published a systematic review with meta-analysis on 6 studies with 176 children with CP demonstrating increased femur BMD after WBV intervention. The efficacy of WBV as a bone anabolic therapy in children with severe motor disabilities appears to be mostly beneficial. However, despite the favorable data reported, there is still not enough evidence to support WBV in clinical practice in children and adolescents with disabilities, which also is in agreement with a recent systematic review [48••].

## Osteogenesis Imperfecta

Osteogenesis imperfecta (OI) is a rare hereditary disease, which can result in extreme bone fragility, limited mobility, and substantial growth deficiency [49]. The majority of patients with OI have a loss-of-function mutation in one of the two genes coding for collagen type I alpha chains, *COL1A1* or *COL1A2*; however, there are also at least 18 other genes that have been associated with OI phenotypes [50]. Pharmacologic treatment regimens with bisphosphonates have successfully been implemented as clinical routine for children with OI to reduce bone resorption, to maximize linear growth, and to reduce the burden of fractures and pain [51]. Bisphosphonates have an approximately decade-long half-life in bone and potential adverse events are still not fully elucidated. Despite treatment, the newly remodeled bone would still comprise defective collagen type I in the classical OI types. WBV has gained some interest as a non-pharmacological anabolic approach for children with OI.

As in WBV intervention studies in children with severe motor disabilities, increased motor function and walking distance have been found as well as an increase in total body BMD (less head) [28]. In contrast, Högler et al. [29] found no significant changes in bone mass. A recent review, in which only 3 eligible studies were found, concluded that WBV intervention could be an alternative option in the management for improving mobility and functional parameters [52].

## Effect of WBV Intervention in Other Groups

There is a large clinical need for further interventional studies about the effects of WBV on bone tissue and bone acquisition in a number of pediatric conditions and syndromes. Positive effects of WBV were demonstrated on all bone mineral content (BMC) and BMD parameters in a randomized controlled trial in individuals with Down syndrome [30]. These findings were supported by a recent review on WBV training, comprising 5 studies including 171 individuals with Down syndrome, which stated that WBV has positive effects on BMD, body composition, and balance [53].

There is an increasing prevalence worldwide of obesity and overweight. WBV intervention has been studied in overweight children; however, there are only two studies regarding the effects of WBV on bone during the last 10 years. One recent randomized study found decreased serum levels of sclerostin after a 12-week WBV intervention in children with obesity, which implies that WBV has direct effects on bone mechanotransduction [12••]. The other study on overweight subjects completed a 10-week WBV intervention, which showed increased BMC and BMD measurements [31]. On the other spectrum of weight disorders, anorexia nervosa is a disease with highly negative effects on bone tissue. One study in females with anorexia nervosa, aged 16 years, showed that daily low-magnitude mechanical stimulation prevented a reduction in bone turnover during bed rest; however, bone mass was not investigated in this study [32].

During the last 10 years, some studies have been published in other disorders or diseases but only as isolated publications with small patients groups, which makes it challenging to summarize the effects of WBV for each disease. In a study including young females with idiopathic scoliosis, the participants used WBV and it proved effective in improving areal BMD at femoral neck and lumbar spine [33]. Single studies exist in hematological diseases such as thalassemia and hemophilia. Fung et al. [34] found that WBV increased total body BMC and areal BMD in a pilot study with adolescent and adult patients with thalassemia. Beneficial effect of WBV training, in terms of increased BMD and quadriceps strength, was also demonstrated in a study on patients with hemophilia [35]. WBV training has also been studied in children recovering from burns who performed regular exercise in conjunction with WBV, which improved leg strength but with reportedly small decreases in some BMC and BMD measurements [36]. Leonard et al. [37] conducted a large WBV intervention in a pediatric cohort of Crohn’s disease and found increased vertebral trabecular BMD, but inconsistent effects on axial and appendicular trabecular volumetric BMD. More and larger clinical studies are clearly needed to draw significant conclusions about the effects of WBV in the described disease groups and other not yet studied populations.

## Perspectives and Concluding Remarks

From this overview of pediatric studies focusing on the last 10 years, it appears that WBV is a safe intervention with few adverse events. WBV, using vibrating platforms of various brands and vibration parameters, has demonstrated beneficial effects on bone mass in some diseases and syndromes in pediatric populations, but definitely not unequivocally in all reported clinical trials. The reported positive effects on muscle strength, balance, and walking speed are more conclusive, in accordance with the mechanostat theory, which in turn could contribute to increased amounts of regular physical activity leading to favorable effects on bone mass and possibly reduced number of fractures. It should be noted that pediatric bone tissue may respond differently in comparison with adult bone since bone tissue is undergoing both modeling and remodeling during longitudinal growth. The response to mechanical stimulation might be different in pediatric bone in contrast to adult bone due to differences in microstructure and mineral-to-collagen ratio.

One of the takeaways of this review is that healthy well-trained children and adolescents, who already perform *high* amounts of physical activity, may not benefit from WBV training since the additive effect does not appear to be further beneficial or intense enough to achieve additional positive effects on skeletal strength. In general, WBV seems to be more beneficial in children and adolescents with low bone mass and reduced activity levels in children with Down syndrome or severe motor disabilities such as CP. The duration for most of the reported WBV studies on bone mass has been rather short (< 6 months), and possibly too short, reflecting the bone modeling/remodeling cycle and to significantly measure a positive net gain in bone mass to access the full potential of WBV. This could partly be explained by practical reasons since WBV interventions are usually quite time-consuming and staff demanding.

The number of reported randomized controlled studies in pediatric populations is clearly inadequate to develop and implement clinical practice guidelines, both in healthy individuals and in most groups of diseases and syndromes. Further and larger prospective studies, longer than 6 months, are still needed to assess the efficacy of WBV on bone mass and bone health in pediatric populations. From a clinical point of view regarding bone health, and in order to make the most of WBV interventions, we also recommend that future research on WBV should focus on exploring optimal vibration parameters (i.e., duration, treatment time, vibration frequency, and peak-to-peak displacements), since reported protocols for these parameters are highly variable. We conclude, from this pediatric review on the last 10 years, that WBV is a safe non-pharmacological anabolic approach to increase bone mass in some pediatric populations.
